# Rate Coefficients for OH + NO (+N_2_) in the Fall-off Regime and the Impact of Water Vapor

**DOI:** 10.1021/acs.jpca.2c02369

**Published:** 2022-06-08

**Authors:** Wenyu Sun, Jos Lelieveld, John N. Crowley

**Affiliations:** Division of Atmospheric Chemistry, Max-Planck-Institute for Chemistry, Hahn-Meitner-Weg 1, 55128 Mainz, Germany

## Abstract

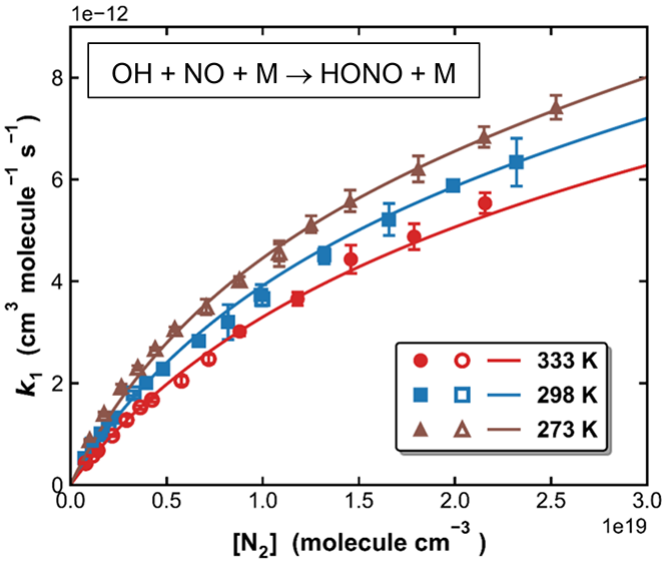

The termolecular, association reaction between OH and NO is a source of nitrous acid (HONO), an important atmospheric trace gas. Rate coefficients for the title reaction as recommended by evaluation panels differ substantially at the temperatures and pressures that prevail in the Earth’s boundary layer where the reaction is in the fall-off regime between low- and high-pressure limiting rate coefficients. Using pulsed laser methods for generation and detection of OH, we have reinvestigated the kinetics of the title reaction at pressures of 22–743 Torr (1 Torr = 1.333 hPa) and temperatures (273, 298, and 333 K) in pure N_2_ and in N_2_–H_2_O bath gases. In situ optical absorption measurements were used to rule out any bias due to NO_2_ or HONO impurities. Our rate coefficients (*k*_1_) in N_2_ bath gas are parametrized in terms of low-pressure (*k*_0_) and high-pressure (*k*_∞_) rate coefficients and a fall-off parameter (*F*_C_) with *k*_1,0_^*N*_2_^ = 7.24 × 10^–31^ (*T*/300 K)^−2.17^ cm^6^ molecule^–2^ s^–1^, *k*_1,∞_ = 3.3 × 10^–12^ (*T*/300 K)^−0.3^ cm^3^ molecule^–1^ s^–1^, and *F*_C_ = 0.53. Used with the “Troe” expression for termolecular reactions, these parameters accurately reproduce the current data in the fall-off regime and also capture literature rate coefficients at extrapolated temperatures. The presence of water vapor was found to enhance the rate coefficients of the title reaction significantly. The low-pressure limiting rate coefficient in H_2_O bath gas is a factor 5–6 larger than in N_2_, at room temperature (*k*_1,0_^*H*_2_*O*^ = 4.55 × 10^–30^ (*T*/300 K)^−4.85^ cm^6^ molecule^–2^ s^–1^) indicating that H_2_O is much more efficient in quenching the association complex HONO* through collisional energy transfer. Based on measurements in N_2_–H_2_O mixtures, a parametrization of *k*_1_ including both N_2_ and H_2_O as third-body quenchers was derived. Neglecting the effect of H_2_O results, e.g., in an underestimation of *k*_1_ by >10% in the tropical boundary layer.

## 1 Introduction

Nitrogen monoxide (NO) is a short-lived intermediate involved in a variety of chemical reactions throughout the Earth’s atmosphere,^1,2^ where it is quickly oxidized to NO_2_ by reaction with O_3_,^3^ peroxy radicals,^4^ NO_3_,^5^ and halogen oxides.^6^ During the day, NO_2_ is rapidly photolyzed back to NO so that a photostationary state between NO and NO_2_ evolves. NO and NO_2_ are together referred to as NO_*x*_, a critical component in the photochemical formation of ozone and smog in the lower atmosphere^1^ and in the destruction of O_3_ in the lower stratosphere.^7^

Both NO and NO_2_ can also be oxidized by reaction with OH in termolecular reactions forming nitrous (HONO) and nitric acid (HNO_3_):
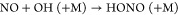
R1
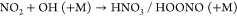
R2During the daytime, HONO is photolyzed to OH + NO with a lifetime of ≥1 h^8^ and may represent a significant source of OH in some environments, especially at sunrise. Apart from its formation in R1, additional sources of HONO include heterogeneous or photochemical reactions of NO*_x_* and other reactive nitrogen compounds on various surfaces, emission from soil, and the photolysis of particulate nitrate.^9−11^

Termolecular reactions, which involve formation of an activated association complex whose relative rate of dissociation back to reactants and collisional quenching determine the effective rate coefficient, are pressure (and temperature) dependent. Such reactions often demonstrate “fall-off” behavior, and the Troe formalism^12^ has been widely adopted to parametrize the rate coefficients in terms of high- and low-pressure limiting rate coefficients (*k*_∞_ and *k*_0_, respectively) and a broadening factor (*F*_C_) to characterize the transition regime in between. Recently, we presented measurements of rate coefficients for the termolecular reaction of OH with NO_2_ and SO_2_ under fall-off conditions at temperatures prevalent from the Earth’s surface to the lower stratosphere.^13−15^

For the title reaction, several experimental data sets^16−32^ were obtained from the 1970s to 1990s, mainly at low pressures in He and Ar bath gases to aid detection of OH. Although highly desirable for the purpose of deriving atmospherically relevant rate coefficients, data sets in N_2_ at conditions relevant for the lower atmosphere (pressures up to 1 bar air) are sparse.^22,27,32^

**Figure 1:**
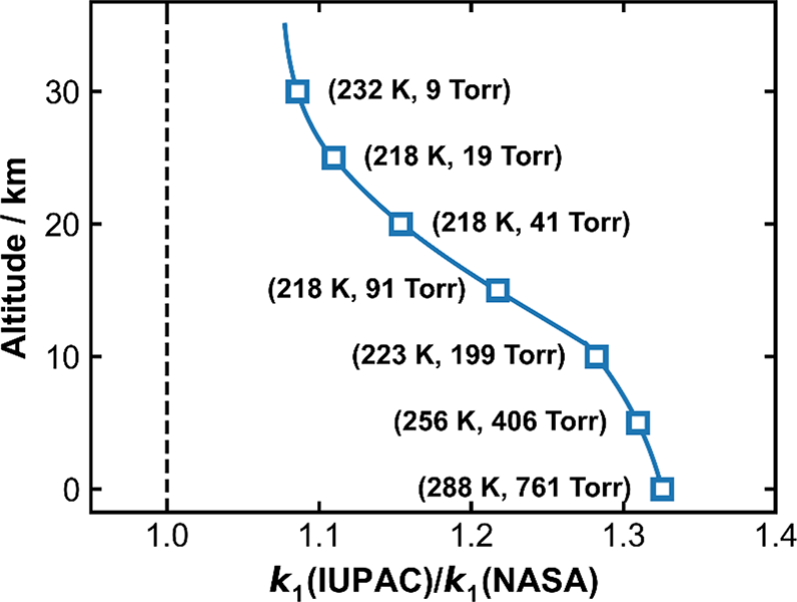
Ratio between rate coefficients, *k*_1_, derived using the IUPAC and NASA parametrizations at different altitudes in the atmosphere. The pressures and temperatures at each altitude were calculated using parameters given in an Earth atmosphere model (https://www.grc.nasa.gov/www/BGH/atmosmet.html).

Figure 1 presents a comparison between values of *k*_1_ recommended by the IUPAC^33,34^ and NASA^35^ evaluation panels at different altitudes in the Earth’s atmosphere (i.e., at different temperatures and pressures). The largest differences are seen for the lower atmosphere (especially in the planetary boundary layer), with better agreement in the stratosphere at low temperatures and pressures. IUPAC and NASA derived similar values of *k*_∞_ (based on high-pressure measurements in He bath gas) and for *k*_0_ based on different studies^19−22,24,26,27^ in which N_2_ was used as a third-body. To some extent, the different rate coefficients can thus be attributed to the broadening factors chosen: 0.6 by NASA and 0.81 by IUPAC.

Previous experimental work in different bath gases^18,19,21,22,25,36^ elucidated the different collisional transfer efficiency of various third-body quenchers for the title reaction. In particular, H_2_O was found to be a more efficient third-body than larger molecules with more vibrational degrees of freedom such as SiF_6_ and CF_4_.^22^ The influence of H_2_O on *k*_1_ was also highlighted in a recent study,^37^ which explored the role of water clusters at very low temperatures (60–135 K) in a Laval nozzle expansion. Our recent studies on the reactions of OH with NO_2_ and SO_2_^14,15^ revealed that HNO_3_/NO_2_ and H_2_SO_4_/SO_2_ ratios in some parts of the atmosphere could be significantly modified by the presence of H_2_O.

The goals of this experimental work are 1) to quantify the impact of H_2_O as a third-body quencher on the title reaction, 2) to derive accurate values of *k*_1_ in the “fall-off” regime in N_2_ bath gas, and 3) to provide a parametrization of *k*_1_ suitable for modeling R1 throughout the atmosphere, thereby reducing uncertainty in this important rate coefficient.

## 2 Experimental Section

The technique of Pulsed-Laser-Photolysis, Laser-Induced Fluorescence setup (PLP-LIF) was employed to determine the rate coefficients for the title reaction under pseudo-first-order conditions where [NO] exceeds [OH] by at least 2 orders of magnitude. The concentration of NO was calculated via manometric methods using accurately diluted gas mixtures. Optical absorption cells were used to monitor potential NO_2_ and HONO impurities in NO mixtures and to measure [H_2_O] in the experiments using H_2_O–N_2_ bath gas.

### 2.1 PLP-LIF Technique

The details of the PLP-LIF setup used in these experiments have been documented in previous publications,^13,38^ and thus, only a brief description is provided here. The reactions took place in a jacketed, cylindrical quartz reactor with a volume of ∼500 cm^3^ the temperature of which was controlled by circulating a 60:40 ethylene glycol–water mixture through an outer jacket. The temperature at the center of the reactor was measured by inserting a J-type thermocouple before and after each experiment. The pressure in the reactor and optical absorption cells (see below) was monitored by capacitance manometers (MKS) with ranges of 100 and 1000 Torr (1 Torr = 1.333 hPa). The experimental pressure was adjusted by varying the total flow rate and pumping speed. The total volume flow rate was varied to maintain an average linear velocity of ∼8–9 cm s^–1^ in the reactor at all experimental temperatures/pressures. The linear velocity at the center of the flow is likely to be larger (by up to a factor of 2 for laminar flow) than 8–9 cm s^–1^, and as the 0.8 mm diameter laser beam propagates at right angles to the gas flow, we can be certain that photolysis occurs in a fresh gas mixture at each laser pulse (operated at 10 Hz).

OH radicals were generated by photolyzing H_2_O_2_ (R3) at a wavelength of 248 nm using a KrF excimer laser (COMPex 205F, Coherent).
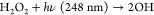
R3OH radicals were excited at 282 nm (A^2^∑ (ν = 1) ← X^2^Π (ν = 0)) by a YAG-pumped dye laser, and the subsequent OH fluorescence was detected by a photomultiplier screened by a 309 nm interference filter and a BG 26 glass cutoff filter. The delay between the triggers of the photolysis and probe lasers was scanned using a digital delay generator. Time-dependent OH profiles (one laser pulse per data point) were obtained by accumulating the fluorescence signals using a boxcar integrator; 20–50 successive profiles were averaged to improve the signal-to-noise ratio. The photolysis laser fluence was measured by a joule meter placed behind the exit window of the reactor, and the shot-to-shot variation in the intensity of the dye laser was monitored by a photodiode. Each OH decay profile was composed of 20 points before the excimer laser was triggered (to determine the background signal) and 100 points after the trigger of the excimer laser for use in deriving the decay kinetics.

### 2.2 Online Optical Absorption Measurements

In our previous studies of atmospherically important, termolecular reactions involving the OH radical,^13,15^ the concentrations of the excess reactants (SO_2_ and NO_2_) were accurately measured through in situ optical absorption techniques. NO displays several resolved absorption features in the VUV^39^ but the more accessible features at 205, 215, and 226 nm are weak and do not coincide with the wavelengths of the atomic line sources available (Hg lines at 185, 254, and 365 nm or Zn at 214 nm) or over the wavelength range (∼230–700 nm) covered by our long-path absorption cell equipped with halogen and deuterium lamps. Compared to NO_2_ and SO_2_, which have affinity for surfaces, NO is easy to handle and has no losses in flow controllers, and diluted samples can be prepared with high accuracy. In this study, the concentration of NO was derived from its partial pressure in a supply canister, its partial flow rate into the reactor, and the total pressure and temperature. The mass flow controllers were freshly calibrated using a Gilibrator.

The purity of the NO sample was checked using an optical absorption cell (*l* = 110 cm) located upstream of the reactor. Light from a deuterium lamp was passed through the cell 8 times (resulting in an optical length of 880 cm) and detected by a low resolution (Δλ = 2 nm) spectrograph (Ocean-Optics USB 2000). Absorption measurements between 250 and 600 nm were inspected for absorption features from NO_2_ and HONO. The minimum absorbance that could be detected was 5 × 10^–4^ at 420 nm, which, using a cross section of 6 × 10^–19^ cm^2^ molecule^–1^^40^ for NO_2_ implies a maximum concentration of 2 × 10^12^ molecules cm^–3^. This is a factor >100 less than the concentration of NO typically used in the experiments (3–20 × 10^14^ molecules cm^–3^) and (as the rate coefficients for reaction with OH are similar) implies that NO_2_ impurity does not significantly bias the loss of OH. Similarly, the characteristic absorption features of HONO at 354, 368, and 384 nm^41^ were not observed, and an upper limit to its concentration could be established, once again excluding a significant bias to the data as a result of the reaction of OH with HONO.

A second (single-pass) optical absorption cell (*l* = 34.8 cm) equipped with a low-pressure 185 nm Hg lamp was located downstream of the reactor to measure water concentrations in the experiments using N_2_–H_2_O bath gases. An absorption cross section of σ_*H*_2_*O*_(185 nm) = 7.14 × 10^–20^ cm^2^ molecule^–1^^42^ was used to retrieve water concentrations, with the pressure and temperature difference between the reactor and the 185 nm cell taken into consideration.

### 2.3 Chemicals

Nitrogen (N_2_, 99.999%) was supplied by Air Liquide and used without further purification. Hydrogen peroxide (H_2_O_2_, AppliChem, 35%) was vacuum distilled to >90 wt % purity. Distilled water (Merck, liquid chromatography grade) was degassed before use. Two different NO–N_2_ mixtures were used for the experiments: one commercial mixture (nominal mixing ratio of 5%) was supplied by Air Liquide, and the other was self-made with 2.75 ± 0.05% NO. The self-made mixture was made using NO (99.9%, purchased from Air Liquide) following fractional distillation to remove impurities such as NO_2_ and other nitrogen oxides. The uncertainty in the mixing ratio is based on a conservative estimate of the accuracy of pressure gauges used to make the mixture.

## 3 Results and Discussion

### 3.1 Rate Coefficients (*k*_1_) in N_2_

**Figure 2:**
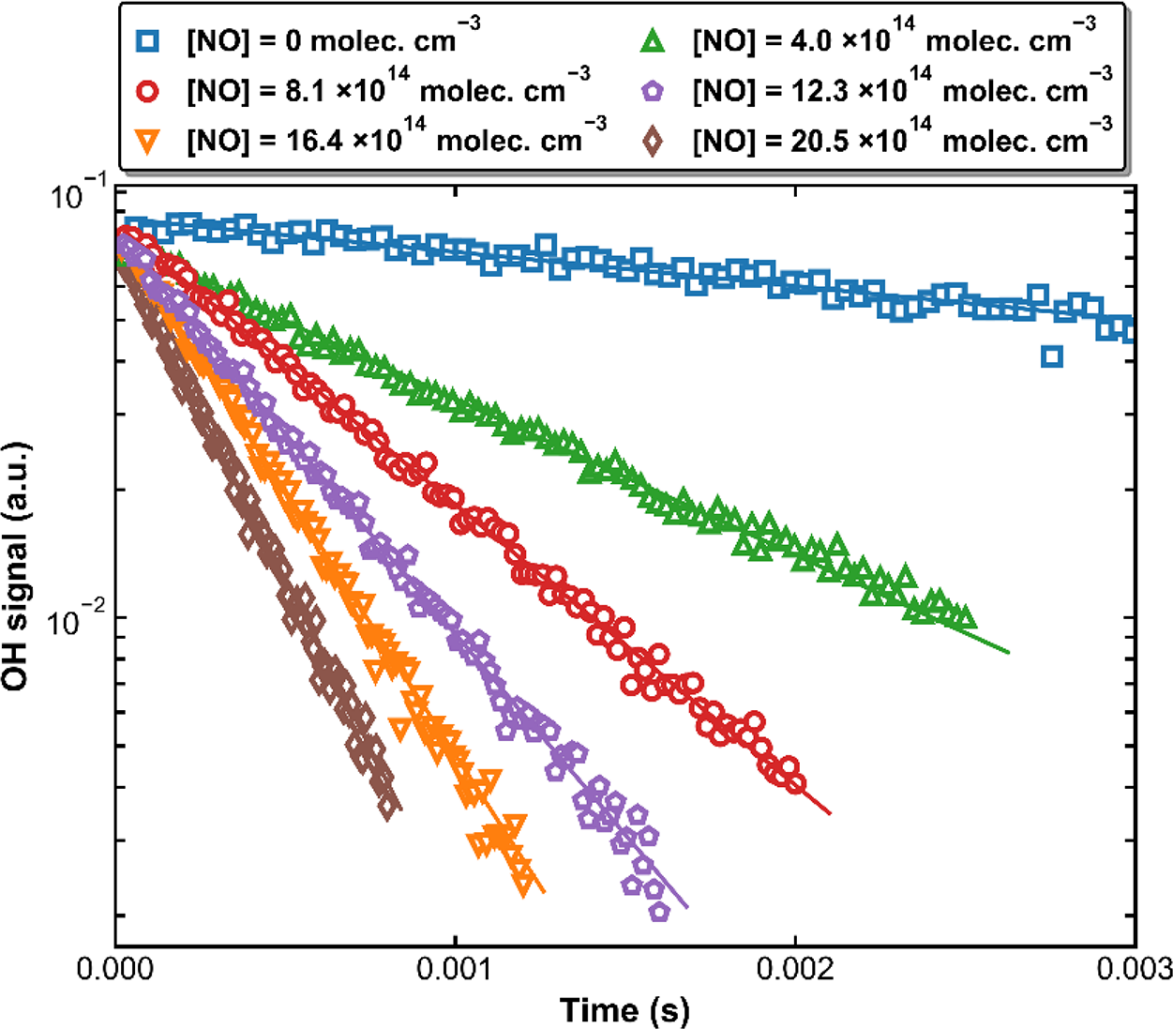
Exponential decay of the OH LIF signal in an experiment at 101.6 Torr N_2_ at 298 K and six different [NO]. The solid lines are the fits to the data using eq 1.

**Figure 3:**
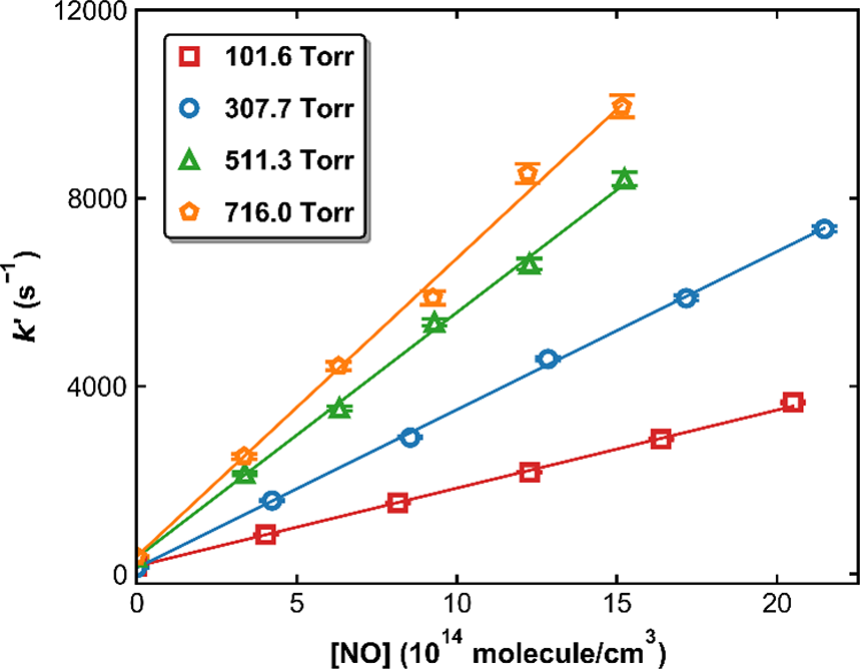
Pseudo-first-order rate coefficients (*k*′) as a function of [NO] at 298 K and four different pressures. The error bars represent 2σ statistical uncertainties. The solid lines are linear regressions according to eq 2.

Rate coefficients for the title reaction in N_2_ were measured at three different temperatures (273, 298, and 333 K) over the pressure range of 22–743 Torr. In all experiments, the OH concentrations were kept sufficiently low (at the level of 10^11^–10^12^ molecules cm^–3^) in comparison to [NO] (3–20 × 10^14^ molecules cm^–3^) to satisfy pseudo-first-order conditions so that the OH decay could be described by
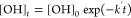
1where [OH]_0_ and [OH]_*t*_ are the OH concentrations at time 0 and *t*, respectively, after the photolysis laser pulse. *k*′ (in s^–1^) is the pseudo-first-order rate coefficient defined as
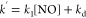
2where *k*_1_ is the bimolecular rate coefficient (in molecules cm^–3^), and *k*_d_ (in s^–1^) accounts for OH removal through diffusion out of the reaction zone and reaction with H_2_O_2_. Figure 2 presents exemplary OH decay profiles at 298 K at different [NO] at a total pressure of ∼100 Torr N_2_. The OH LIF signals decay exponentially, and the fits to eq 1 yield the corresponding values of *k*′. Figure 3 plots *k*′ versus [NO] at four different pressures; *k*_1_ is derived from the linear regression of *k*′ versus [NO] according to eq 2. Values of *k*_1_, together with the statistical (2σ) standard errors, are summarized in Table 1, in which the experimental conditions are also provided. We estimate the potential systematic error (mainly in [NO]) as <5% as the NO–N_2_ mixture was prepared as precisely as possible, and all the flow controllers were calibrated prior to the experiments. Overall, an uncertainty of 8% was estimated for *k*_1_.

**Table 1:** Values of *k*_1_ Measured in N_2_ Bath Gas^a^

*T* (K)	*p* (Torr)	[M]	flow rate (SCCM)	[NO]	*k*_1_	NO mixture
273	28	0.99	295	3.12–15.86	0.88 ± 0.02	b
273	49.1	1.74	452	2.98–13.51	1.39 ± 0.03	a
273	49.1	1.74	454	3.55–18.04	1.40 ± 0.05	b
273	74.6	2.64	688	3.56–18.08	1.92 ± 0.06	b
273	98.7	3.49	893	3.63–18.45	2.30 ± 0.05	b
273	124.4	4.40	1220	3.35–17.02	2.67 ± 0.03	b
273	153.3	5.42	1406	3.58–18.20	3.06 ± 0.05	b
273	199.4	7.05	1777	3.68–18.72	3.50 ± 0.16	b
273	248.3	8.78	2148	3.79–19.29	4.02 ± 0.08	b
273	306.7	10.85	2538	3.32–15.02	4.54 ± 0.25	a
273	306.7	10.85	2540	3.96–20.15	4.57 ± 0.18	b
273	353.8	12.51	3010	3.23–14.61	5.12 ± 0.16	a
273	411.3	14.54	3371	3.35–15.17	5.58 ± 0.21	a
273	511.4	18.08	4203	3.34–15.13	6.21 ± 0.25	a
273	608.1	21.50	5033	3.32–15.02	6.84 ± 0.20	a
273	714	25.25	5892	3.33–15.06	7.42 ± 0.23	a
298	22.2	0.72	182	3.03–13.86	0.53 ± 0.04	a
298	35.2	1.14	307	3.48–17.55	0.79 ± 0.03	b
298	50.2	1.63	436	3.46–17.58	1.00 ± 0.03	b
298	50.2	1.63	435	2.90–13.14	1.00 ± 0.01	a
298	61.3	1.99	504	3.66–18.60	1.20 ± 0.02	b
298	71.4	2.31	621	2.88–13.09	1.32 ± 0.02	a
298	100.2	3.25	821	3.07–13.90	1.79 ± 0.05	a
298	101.6	3.29	990	3.71–18.87	1.80 ± 0.05	b
298	121.2	3.93	1020	2.99–13.53	2.00 ± 0.03	a
298	148.6	4.81	1284	2.88–13.13	2.28 ± 0.08	a
298	205.9	6.67	1652	3.12–14.19	2.83 ± 0.03	a
298	253	8.20	2016	5.75–20.93	3.20 ± 0.34	a
298	305.9	9.91	2378	3.24–14.65	3.73 ± 0.21	a
298	307.7	9.97	2380	3.89–19.77	3.65 ± 0.14	b
298	407.5	13.20	3100	3.31–14.97	4.51 ± 0.16	a
298	511.3	16.56	3823	3.37–15.23	5.22 ± 0.32	a
298	614	19.89	4426	3.40–15.38	5.88 ± 0.06	a
298	716	23.19	5388	3.35–15.15	6.34 ± 0.47	a
333	26.9	0.78	235	3.09–15.68	0.43 ± 0.01	b
333	26.9	0.78	232	2.59–11.80	0.43 ± 0.04	a
333	39.4	1.14	275	3.86–19.63	0.58 ± 0.01	b
333	48.6	1.41	394	3.32–16.86	0.68 ± 0.02	b
333	48.6	1.41	392	2.78–12.64	0.71 ± 0.02	a
333	74.7	2.17	594	3.39–17.21	0.97 ± 0.02	b
333	100.6	2.92	753	3.59–18.27	1.28 ± 0.08	b
333	124.9	3.62	963	3.49–17.74	1.52 ± 0.04	b
333	145.9	4.23	1245	3.15–16.03	1.67 ± 0.04	b
333	198.4	5.75	1615	2.77–12.52	2.05 ± 0.06	a
333	198.8	5.76	1627	3.29–16.72	2.05 ± 0.09	b
333	248	7.19	2008	3.32–16.89	2.48 ± 0.10	b
333	303.3	8.79	2277	3.00–13.57	3.02 ± 0.09	a
333	407.3	11.81	3029	3.03–13.71	3.66 ± 0.13	a
333	502.7	14.57	3742	3.03–13.69	4.43 ± 0.28	a
333	616.2	17.86	4486	3.10–14.00	4.88 ± 0.25	a
333	743.8	21.56	5241	3.20–14.46	5.54 ± 0.20	a

a Units of [M] are 10^18^ molecules cm^–3^. Units of *k*_1_ are 10^–12^ cm^3^ molecule^–1^ s^–1^. Units of [NO] are 10^14^ molecules cm^–3^. The given total flow rates are calibrated values. Mixtures “a” and “b” are the self-made NO–N_2_ mixture and the 5% NO in the N_2_ mixture supplied by Air Liquide, respectively.

**Figure 4:**
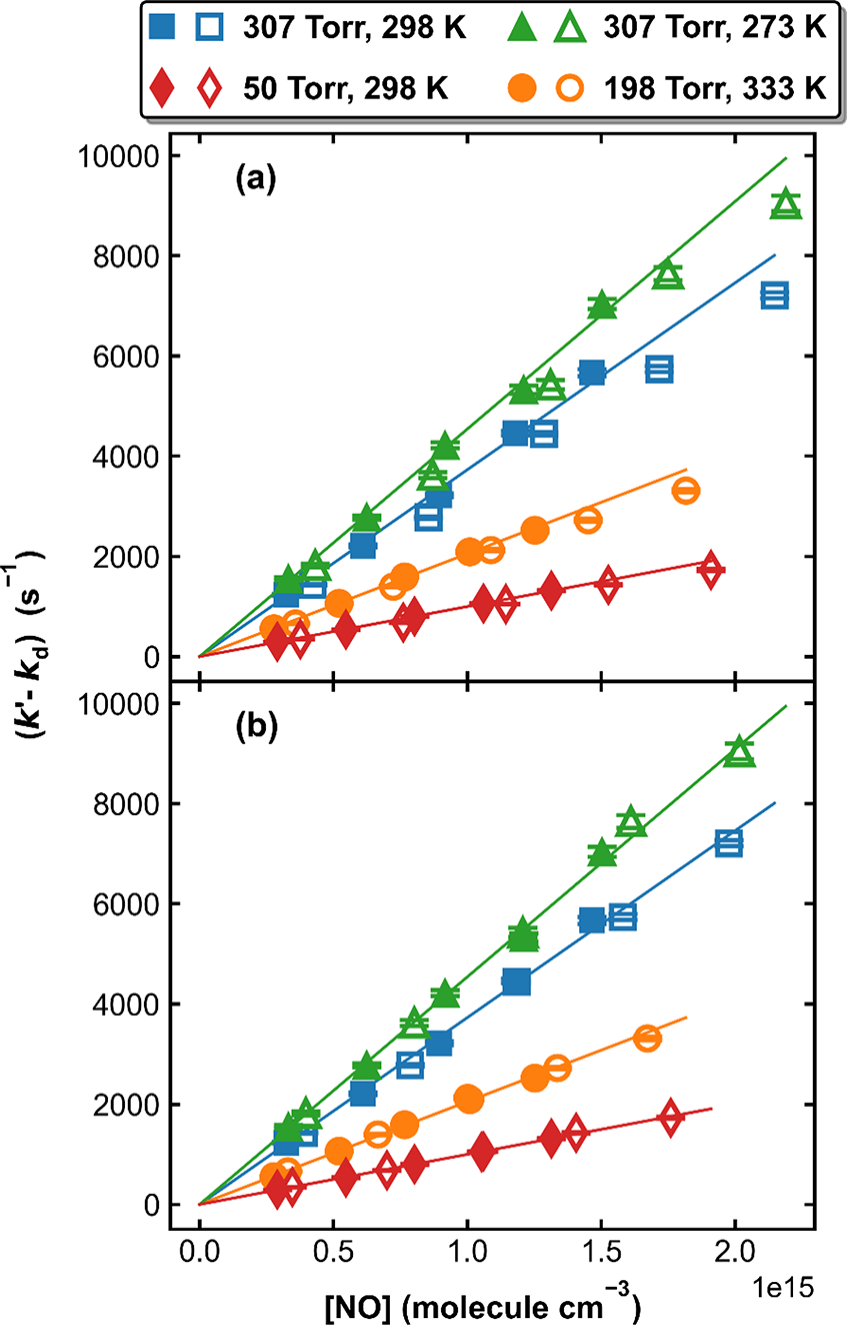
Measured values of (*k*′-*k*_d_) as a function of [NO] using the self-made mixture (closed symbols) and the commercial mixture (open symbols) under four different experimental conditions. The solid lines are linear regressions of measurements with the self-made mixture. The NO mixing ratio is 5% in (a) and corrected to 4.60% in (b).

**Figure 5:**
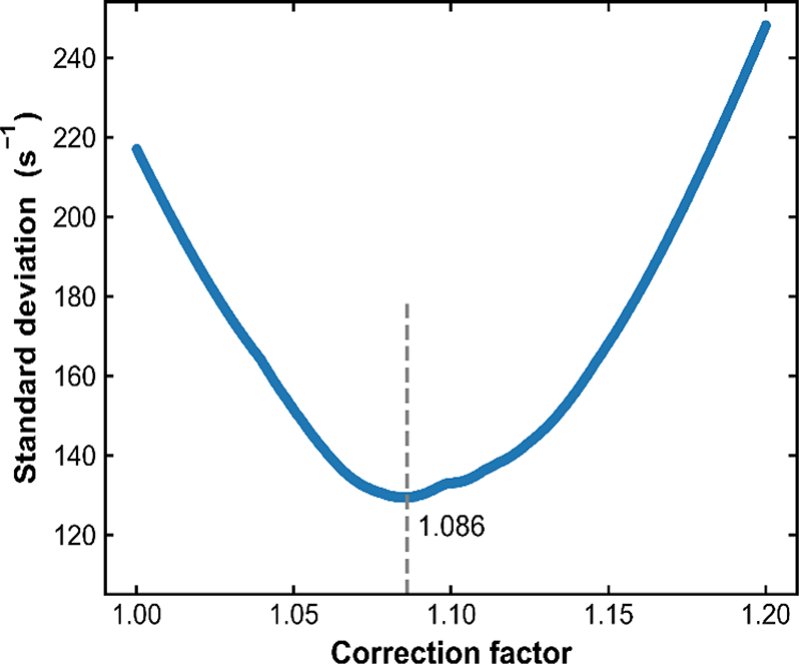
Standard deviation for the difference between the (*k*′-*k*_d_) measurements with the commercial (nominal 5%) NO mixture (the open symbols in Figure 4) and the linear regressions (solid lines in Figure 4) through data points obtained with the self-made NO mixture as a function of the correction factor for the NO mixing ratio in the commercial sample.

As mentioned in the Experimental Section, two NO–N_2_ mixtures were used for the measurements. The first set of experiments was carried out using the bottled, commercial mixture, and the second set was carried out using our self-made mixture. The commercial mixture was not a primary standard, and thus the mixing ratio of NO was not sufficiently well-known to derive accurate rate coefficients. To obtain the exact NO concentration in the commercial (nominally 5%) mixture, measurements were performed under identical conditions using the two mixtures. Values of (*k*′-*k*_d_), are plotted as a function of [NO] in Figure 4(a), in which the closed and open symbols represent measurements using the self-made and the commercial mixtures, respectively. The solid lines are the linear regressions for the (*k*′-*k*_d_) measurements (in s^–1^) with the self-made 2.75% NO mixture, which lie consistently above the data points obtained using the commercial mixture, indicating that the true NO concentration in the Air Liquide bottle should be lower than the nominal value. By systematically varying the mixing ratio of the commercial sample (using correction factors between 1 and 1.2) and refitting the data, we derived the best fit to the entire data set (i.e., the minimum standard deviation in the difference between the open symbols and solid lines in Figure 4). As shown in Figure 5, a correction factor of 1.086 (i.e., the true NO mixing ratio in the commercial sample is 4.60%) gives the best result. Figure 4(b) plots (*k*′-*k*_d_) for all data obtained under identical conditions (both NO samples) when this correction is applied.

**Figure 6:**
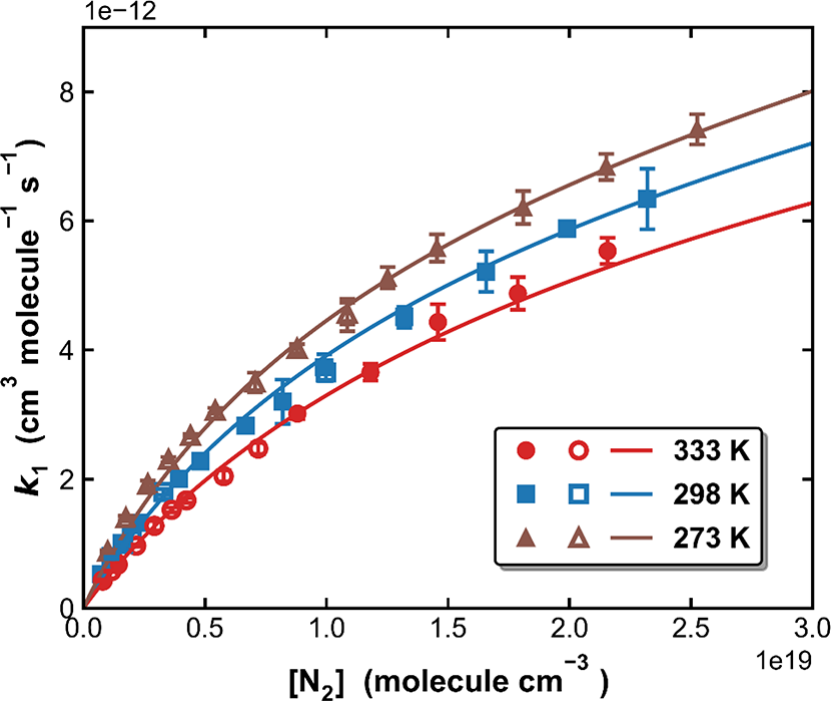
Measured *k*_1_ (symbols) as a function of [N_2_] at 273, 298, and 333 K in this work. The closed and open symbols represent measurements using the self-made and the Air Liquide mixtures, respectively. The solid lines are the fits (*Method 4*) of experimental data to eqs 3 and 4 with *k*_1,0_^*N*_2_^ = 7.24 × 10^–31^ cm^6^ molecule^–2^ s^–1^, *n* = 2.17, *k*_1,∞_ = 3.30 × 10^–11^ cm^3^ molecule^–1^ s^–1^, *m* = 0.3, and *F*_C_ = 0.53.

Figure 6 displays values of *k*_1_ measured in N_2_ bath gas as a function of the N_2_ concentration (N_2_ pressure was 22–744 Torr) at three different temperatures (273, 298, and 333 K). The solid lines are global, least-squares fits according to the Troe formalism^12^ for termolecular reactions
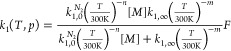
3where *k*_1,0_^*N*_2_^ (in cm^6^ molecule^–2^ s^–1^) and *k*_1,∞_ (in cm^3^ molecule^–1^ s^–1^) are the high-pressure and low-pressure limiting rate coefficients, respectively; *T* is the temperature in Kelvin; [*M*] is the molecular density in molecules cm^–3^; and *n* and *m* are dimensionless temperature exponents. The broadening factor *F* accounts for the lower rate coefficients in the fall-off regime compared to predictions by the Lindemann–Hinshelwood mechanism and is expressed as
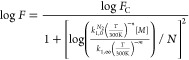
4where *N* = 0.75–1.27 log *F*_C_, and *F*_C_ is the broadening factor at the center of the fall-off curve.

To reduce the number of fit variables, and also because a relatively small temperature range is covered by the current measurements, we fix *k*_1,∞_ and its temperature dependence to values obtained in experiments in He at pressures up to 150 bar^28^ that indicated that *k*_1,∞_ is ∼3 × 10^–11^ cm^3^ molecule^–1^ s^–1^ with the temperature dependence (*m* = 0.3) derived from measurements at 250, 298, and 400 K.^30^ Hence, only the parameters *k*_1,0_^*N*_2_^, its temperature dependence (*n*), and *F*_C_ are allowed to vary.

The results are summarized in Figure 6 (solid lines) and in Table 2 where we also list the values preferred by IUPAC and NASA. In the Supporting Information, we also list and discuss the results obtained when different (or no) constraints to the fits are used. In summary, the fits obtained when fixing *k*_1,∞_ or when freely varying all parameters are of similar quality. However, the values of *k*_1,∞_, derived by freely varying all parameters are significantly lower than the results of high pressure experiments and have a strong negative temperature dependence, which reflects the fact that our data (in the fall-off region) do not define the high-pressure limiting rate coefficient well. The value of *k*_1,0_^*N*_2_^ = 7.24 × 10^–31^ (*T*/300 K)^−2.17^ cm^6^ molecule^–2^ s^–1^ that we obtain is in good agreement with those preferred by IUPAC and NASA (see Table 2), although the value of *F*_C_ = 0.53 is substantially lower than the calculated value of 0.81. We note that fixing *F*_C_ to 0.81 and using the IUPAC parameters for *k*_1,∞_ and *m* preclude a good fit to our data set (see discussion in the SI).

**Table 2:** Parametrization of *k*_1_ in N_2_

	*k*_1,0_^*N*_2_^^a^	*n*	*k*_1,∞_^b^	*m*	*F*_C_	temp (K)
this work	7.24	2.17	**3.3**	**0.3**	0.53	273–333
IUPAC	7.4	2.4	3.3	0.3	0.81	200–400
NASA^c^	7.1	2.6	3.6	0.1	0.6	--

a Units of 10^–31^ cm^6^ molecule^–2^ s^–1^.

b Units of 10^–12^ cm^3^ molecule^–1^ s^–1^.

c The simplified form of the Troe expression for termolecular reactions used by NASA can be found in the Supporting Information. Numbers in bold type were fixed during fitting.

### 3.2 Comparison with Previous Measurements and Parametrizations for N_2_ Bath Gas

**Figure 7:**
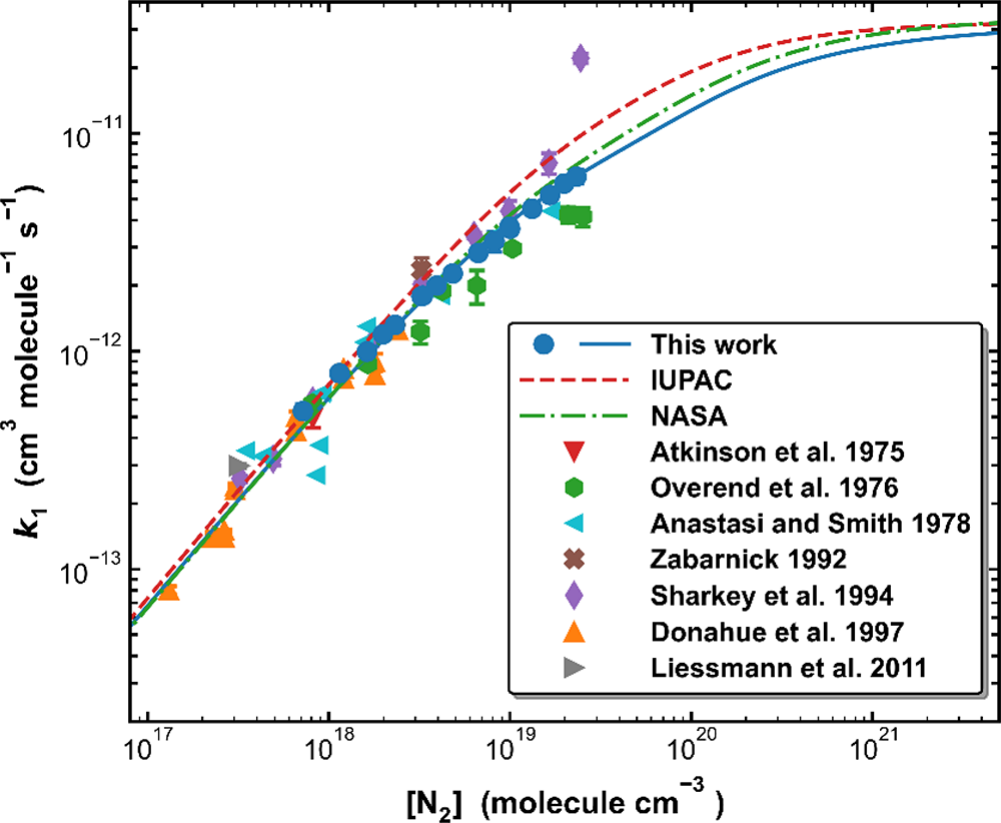
A comparison of measured and parametrized values of *k*_1_ in N_2_ bath gas at 298 K. The lines are values of *k*_1_ derived from the parametrizations presented in this work (*Method 4*) and those by the IUPAC and NASA data-evaluation panels.

Figure 7 presents a comparison of the present and previous measurements of *k*_1_ in N_2_ at around 298 K, our parametrization (Table 3) and the IUPAC and NASA evaluations at the same temperature.

**Table 3:** Values of *k*_1_ Obtained in N_2_–H_2_O Bath Gases

*T* (K)	*p* (Torr)	M^a^	[H_2_O]^b^	*x*_*H*_2_*O*_	*x*_*N*_2__	*k*_1_^c^
273	50.0	1.77	0.00	0.000	1.000	1.30 ± 0.04
	50.3	1.78	0.24	0.013	0.987	1.37 ± 0.03
	50.4	1.78	0.44	0.024	0.976	1.43 ± 0.00
	49.9	1.76	0.77	0.044	0.956	1.49 ± 0.05
	50.4	1.78	0.95	0.054	0.946	1.52 ± 0.06
	50.2	1.77	1.17	0.066	0.934	1.69 ± 0.04
	50.1	1.77	1.43	0.081	0.919	1.72 ± 0.05
298	50.2	1.63	0.00	0.000	1.000	1.00 ± 0.03
	50.5	1.63	0.26	0.016	0.984	1.02 ± 0.01
	50.0	1.62	0.49	0.031	0.969	1.04 ± 0.01
	50.2	1.63	0.56	0.035	0.965	1.12 ± 0.04
	50.0	1.62	0.91	0.056	0.944	1.16 ± 0.02
	50.1	1.62	1.20	0.074	0.926	1.28 ± 0.03
	49.9	1.62	1.55	0.096	0.904	1.33 ± 0.08
	50.3	1.63	2.04	0.125	0.875	1.40 ± 0.06
	50.3	1.63	2.51	0.154	0.846	1.48 ± 0.06
	50.4	1.63	2.68	0.164	0.836	1.44 ± 0.03
	50.1	1.62	2.94	0.181	0.819	1.60 ± 0.05
	49.9	1.62	3.26	0.202	0.798	1.61 ± 0.13
	49.8	1.61	3.74	0.232	0.768	1.72 ± 0.05
	49.9	1.62	4.02	0.249	0.751	1.88 ± 0.12
333	49.8	1.44	0.00	0.000	1.000	0.71 ± 0.01
	49.9	1.45	0.37	0.026	0.974	0.78 ± 0.04
	49.6	1.44	0.82	0.057	0.943	0.84 ± 0.01
	49.8	1.44	1.17	0.081	0.919	0.92 ± 0.04
	50.4	1.46	1.54	0.105	0.895	0.94 ± 0.06
	50.3	1.46	2.10	0.144	0.856	1.00 ± 0.09
	50.0	1.45	2.60	0.180	0.820	1.06 ± 0.08
	50.3	1.46	3.06	0.210	0.790	1.10 ± 0.07
	50.0	1.45	3.35	0.232	0.768	1.21 ± 0.09

a Units are 10^18^ molecules cm^–3^.

b Units are 10^17^ molecules cm^–3^.

c Units are 10^–12^ cm^3^ molecule^–1^ s^–1^

Over the fall-off regime, most literature data sets obtained in N_2_ were obtained at pressures well below 1 bar.^22,23,27,29^ The current measurements and parametrization agree well with the data from Anastasi and Smith^23^ and Donahue et al.,^29^ while the data sets reported in Overend et al.^22^ and Sharkey et al.^27^ lie slightly below and above our measurements, respectively, at pressures >100 Torr. We further compared our parametrized rate coefficients to literature data obtained at temperatures beyond the current experimental range of 273–333 K. Data has been reported at 233 and 405 K (Anastasi and Smith^23^) and 216 K (Sharkey et al.^27^), and both our new parametrization and the NASA evaluation reproduce the measurements of *k*_1_ at 233 and 405 K, while the IUPAC parametrization results in higher values, especially at 233 K (Figure S6). The rate coefficients reported by Sharkey et al.^27^ at 216 K are larger than the parametrized rate coefficients, and their values at 298 K are also larger than reported in all other data sets (see Figure 7), which indicates a systematic bias related to their determination of the NO concentration.

Figure 7 (and Figure S5) shows that the parametrization derived in this work converges with those of the evaluation panels, particularly NASA, at low pressures.^23,27,29^ Values of *k*_1,0_ derived at low pressures using the discharge flow technique^19−21,24^ vary greatly (from 5.8 × 10^–31^ to 15 × 10^–31^ cm^6^ molecule^–2^ s^–1^) which might be related to experimental difficulties including, e.g., correcting for OH wall losses and axial diffusion, and these data are not represented in Figure 7.

### 3.3 Influence of Water Vapor on *k*_1_

Two recent publications from this group on termolecular reactions of OH indicated that H_2_O is a very efficient collision partner compared to N_2_.^14,15^ We therefore measured *k*_1_ in N_2_–H_2_O bath gases at a total pressure of 50 Torr and three different temperatures (273, 298, and 333 K). The relatively low pressure was chosen to best separate the contributions of H_2_O and N_2_ and remains far from the limiting high pressure regime. The H_2_O mixing ratio *x*_*H*_2_*O*_, defined as the molar fraction of H_2_O in the N_2_ bath gas, was varied, and the corresponding values of *k*_1_ were measured. *x*_*H*_2_*O*_ was kept below 10% at 273 K and 25% at 298 and 333 K to avoid condensation of water in any part of the reactor or optical cell. In all experiments, the fluctuation of the total pressure was <1% so that the resulting influence on the measured *k*_1_ was less than 1%.

**Figure 8:**
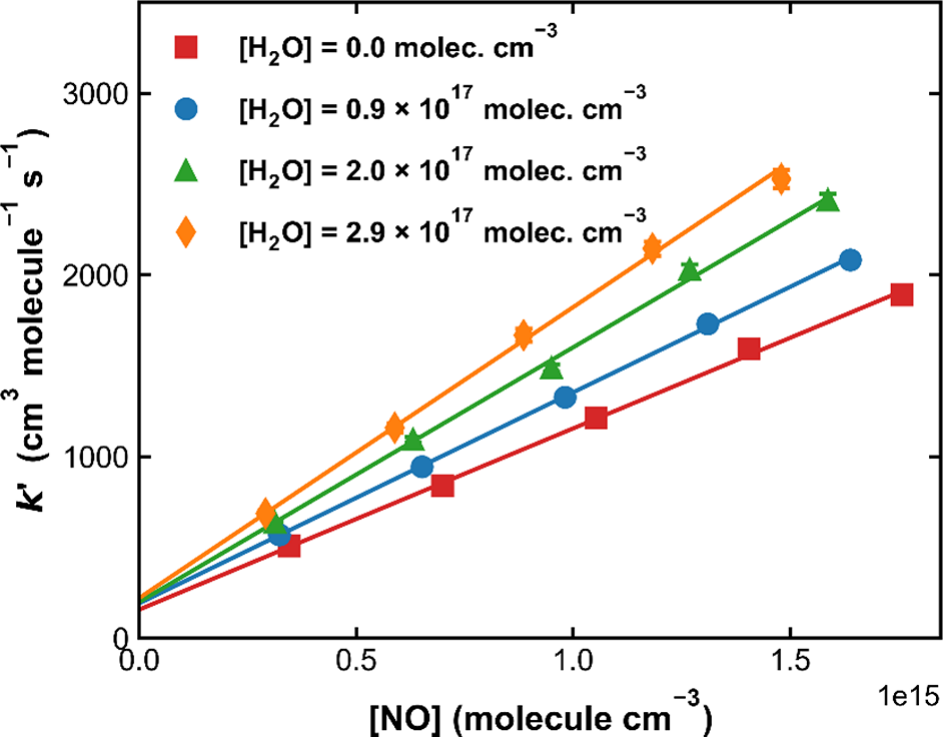
*k*′ as a function of [NO] in N_2_–H_2_O bath gases with different water concentrations at 298 K and a total pressure of 50 Torr. The linear lines are the corresponding linear regressions.

Figure 8 plots values of *k*′ as a function of the NO concentration in four bath gases containing different amounts of water vapor at 298 K and documents an increase in the slope of the linear regression (i.e., in *k*_1_), with the concentration of water. At the highest water vapor concentration used (2.9 × 10^17^ molecules cm^–3^), *k*_1_ increases by around 60% compared to the value obtained in pure N_2_ at this pressure and temperature.

**Figure 9:**
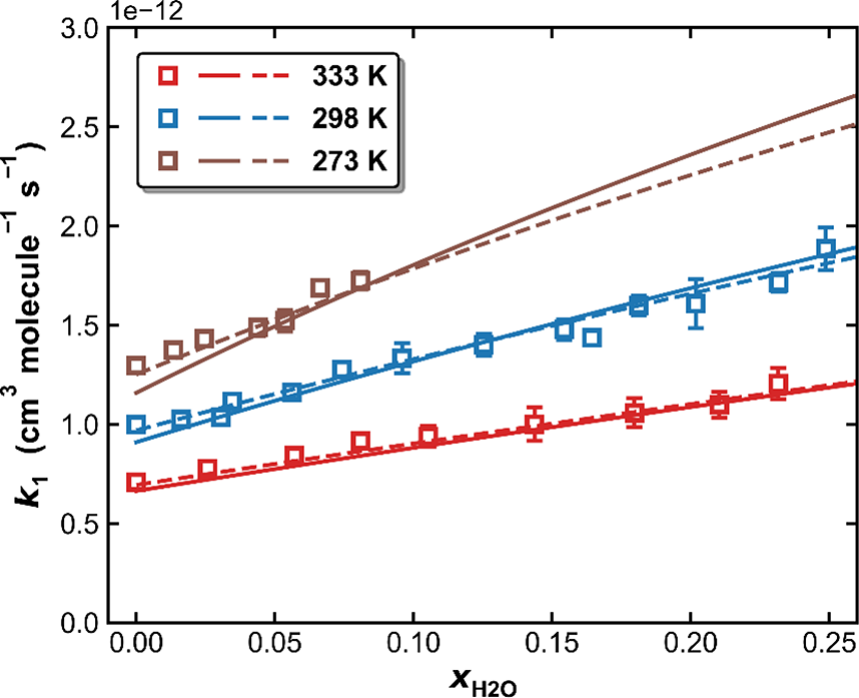
*k*_1_ as a function of *x*_*H*_2_*O*_ in N_2_–H_2_O bath gases at a total pressure of 50 Torr and three different temperatures. The symbols are measurements. The solid lines are fits to eq 5 and eq 6 with *k*_1,0_^*N*_2_^, *n* = 2.17, *k*_1,∞_, *m*, and *F*_C_ constrained using parameters obtained in *Method 4* (Table S1). The resulting parameters in H_2_O bath gas are *k*_1,0_^*H*_2_*O*^ = 4.55 × 10^–30^ cm^6^ molecule^–2^ s^–1^ and *o* = 4.85. The dashed lines are the corresponding fits when using *k*_1,0_^*N*_2_^, *n* = 2.17, *k*_1,∞_, *m*, and *F*_C_ constrained using parameters obtained in *Method 1* (Table S1). The resulting parameters in H_2_O bath gas are *k*_1,0_^*H*_2_*O*^ = 3.81 × 10^–30^ cm^6^ molecule^–2^ s^–1^ and *o* = 4.19.

Values of *k*_1_ obtained in N_2_–H_2_O bath gases at 50 Torr and at three different temperatures are plotted against *x*_*H*_2_*O*_ in Figure 9. The increasing value of *k*_1_ with *x*_*H*_2_*O*_ indicates that H_2_O is a more efficient third-body quencher than N_2_ for the title reaction and the effect of water on *k*_1_ is also dependent on the temperature (largest slope at the lowest temperature). To evaluate the role of water in OH + NO kinetics and to derive a parametrization for *k*_1_, the following equations are used to analyze the data
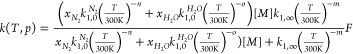
5where *x*_*H*_2_*O*_ and *x*_*N*_2__ are the mole fractions of H_2_O and N_2_, *k*_1,0_^*H*_2_*O*^ is the low-pressure limiting rate coefficient (cm^6^ molecule^–2^ s^–1^) in pure H_2_O, and *o* is a dimensionless temperature exponent. The broadening factor *F* is now defined as
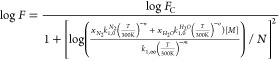
6Equation 5 is essentially an extension of eq 3 in which the low-pressure limiting rate coefficients in N_2_ and H_2_O are linearly mixed. In eq 6, the same *F*_C_ is assumed for both N_2_ and H_2_O bath gases for simplification purposes.^14^

Adopting the “dry” parameters obtained in pure N_2_ (*k*_1,0_^*N*_2_^, *n*, *k*_1,∞_, *m*, and *F*_C_) using *Method 1* or *Method 4* (listed in the first and fourth row of Table S1), a global, least-squares fit to the N_2_/H_2_O data set results in *k*_1,0_^*H*_2_*O*^ = 3.81 × 10^–30^ (*T*/300 K)^−4.19^ cm^6^ molecule^–2^ s^–1^ (*Method 1*, dashed lines in Figure 9) or *k*_1,0_^*H*_2_*O*^ = 4.55 × 10^–30^ (*T*/300 K)^−4.85^ cm^6^ molecule^–2^ s^–1^ (*Method 4*, solid lines in Figure 9). While the differences in the fits obtained using *Method 1* and *Method 4* are slight at 333 and 298 K, the use of *Method 1* results in a poorer fit to the data at 273 K, which is (at least partially) due to the use of a larger value of *k*_∞_. For the purpose of constraining the fit to the data of the H_2_O–N_2_ experiments, the accurate characterization of *k*_1_ at low pressures is of primary importance, and the correct derivation of *k*_1,∞_ is less essential. As the rate coefficients at 50 Torr are far from *k*_1,∞_ and because the use of parameters obtained using *Method 1* to constrain the fit gives the best fit, we prefer *k*_1,0_^*H*_2_*O*^ = 3.81 × 10^–30^ (*T*/300 K)^−4.19^ cm^6^ molecule^–2^ s^–1^.

In both cases, it is clear that *k*_1,0_^*H*_2_*O*^ (300 K) is a factor 5–6 larger than *k*_1,0_^*N*_2_^ (300 K), similar to the results obtained in our studies of OH + NO_2_ (+M) and OH + SO_2_ (+M).^14,15^

**Figure 10:**
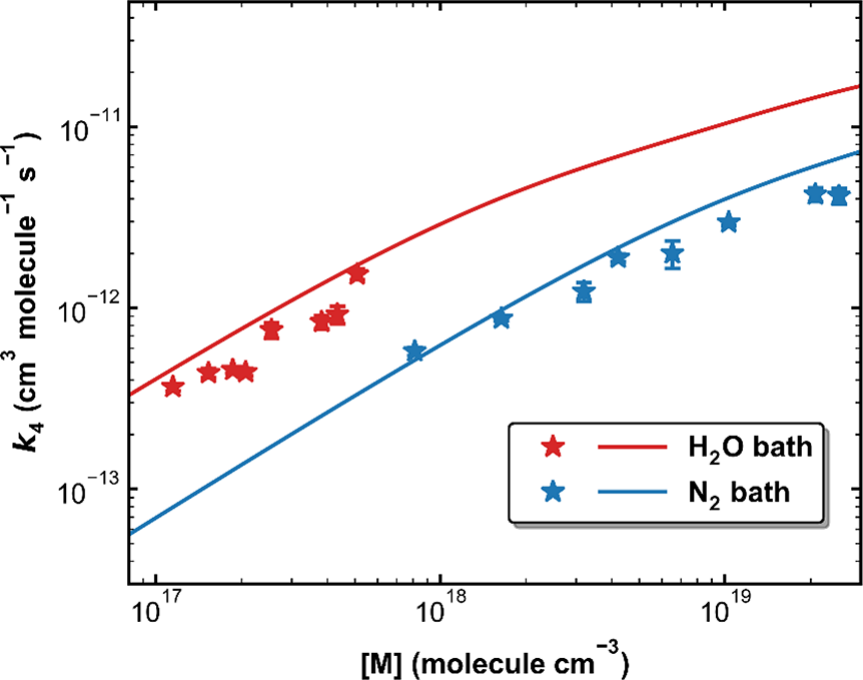
Fall-off curves for *k*_1_ in H_2_O and N_2_ bath gases at 295 K. Solid lines are the current parametrizations based on *Method 4* (see Table S1). Symbols are measurements reported by Overend et al.^22^

Overend et al.^22^ performed measurements in He–H_2_O mixtures where the H_2_O partial pressure ranged from 3 to 16 Torr over a total pressure of 20–30 Torr at 295 K. The results are displayed in Figure 10 which also plots our parametrized fall-off curves for *k*_1_ in pure H_2_O and pure N_2_ for comparison. In both bath gases, the current data and parametrizations lie above the rate coefficients reported by Overend et al.^22^ whose data are significantly more scattered than those of the present study, which appears to stem from scatter in the plots of *k*′ versus [NO]. Overend et al.^22^ analyzed their data with a two-step Lindeman scheme and concluded that the collisional energy transfer efficiency of H_2_O was a factor 8.3 greater than that of N_2_, somewhat larger than the value of 5–6 derived in this work.

Liessmann et al.^37^ addressed the role of H_2_O in their studies of the title reaction in a Laval-nozzle expansion (61–135 K) at pressures close to 1 Torr and documented a significant increase in the rate coefficient (factors of 1.06 to 1.44) in the presence of H_2_O (at 3% of the total pressure). Such a large enhancement in the rate coefficient in the presence of just 3% H_2_O (i.e., *x*_*H*_2_*O*_ = 0.03) is much greater than observed at the higher temperatures of the present study or than of Overend et al.^22^ As discussed by Liessmann et al.,^37^ the supersaturation of H_2_O in the expansion favors cluster formation and the formation of OH(H_2_O)_*n*_, NO(H_2_O)_*n*_ prior to reaction, and also formation of the cluster HONO(H_2_O)_*n*_ may play a role in their experiments and explain the much larger effects they observed. In contrast to the Laval-nozzle experiments, low temperatures in the Earth’s atmosphere are accompanied by low water–vapor mixing ratios, and the results obtained in the present study (and in that of Overend et al.^22^) are relevant for estimating the impact of considering (or, conversely, neglecting) the enhancement of *k*_1_ in the presence of H_2_O.

### 3.4 Implications for the Atmosphere

The discussion above indicates that H_2_O is a much more efficient third-body quencher than N_2_ for the NO + OH reaction, and a simple calculation serves to illustrate the impact of water vapor on the rate coefficient of the title reaction in the atmosphere. Consider the tropical boundary layer with a typical temperature of 30 °C (303 K), a total pressure of 1 bar (750 Torr), and a humidity of 100%. The major components (bath gases) of the air are 567 Torr N_2_, 151 Torr O_2_, and 32 Torr H_2_O. We assume that O_2_ has the same quenching efficiency as N_2_, which is generally a very good approximation. Despite its lower concentration, the higher quenching efficiency of H_2_O contributes more than O_2_ to the collisional relaxation of HONO* (and thus the rate coefficient). The current parametrization yields values of *k*_1_ (1 bar, 303 K) = 6.17 × 10^–12^ cm^3^ molecule^–1^ s^–1^ if the impact of H_2_O is ignored and a >10% larger value of 6.86 × 10^–12^ cm^3^ molecule^–1^ s^–1^ when H_2_O is considered (using *k*_1,0_^*H*_2_*O*^ = 3.81 × 10^–30^ (*T*/300 K)^−4.19^ cm^6^ molecule^–2^ s^–1^). At the same temperature and pressure, the parametrizations of the IUPAC and NASA panels (neither of which takes H_2_O into account) result in values of 9.36 × 10^–12^ and 7.09 × 10^–12^ cm^3^ molecule^–1^ s^–1^, respectively. The present data set and parametrization should be used to reassess the kinetic data for the title reaction and guide the IUPAC and NASA panels toward reaching consensus on their preferred values, especially at lower altitudes.

## 4 Conclusions

Rate coefficients of the title reaction NO + OH were measured at various temperatures and pressures (N_2_) in the fall-off regime and used to develop a parametrization that accurately describes the present data and literature data sets even at temperatures outside the range of our measurements. Experiments in N_2_–H_2_O bath gases showed that water is a more efficient third-body quencher than N_2_ by a factor of 5–6. The water effect was parametrized using a Troe type expression considering multiple bath gas components, which provides a comprehensive and reliable basis for atmospheric modeling.

## Supporting Information Available

Description of fitting constraints (Methods 1–5) and associated figures (Figures S1–S4), comparison of *k*_1_ with evaluations (Figures S5 and S6), NASA parametrization method for termolecular reactions, and parametrization of *k*_1_ in N_2_–H_2_O bath gases using different values of *F*_C_ for N_2_ and H_2_O (PDF)
