# Supplementary material for: Rate Coefficients for OH + NO (+N2) in the Fall-off Regime and the Impact of Water Vapor
Source: J Phys Chem A. 2022 Jun 8;126(24):3863–72. doi: 10.1021/acs.jpca.2c02369 (PMC9234955; doi:10.1021/acs.jpca.2c02369)
Supplement: Supplementary file 1 — jp2c02369_si_001.pdf [file jp2c02369_si_001.pdf]

Supporting Information for Publication

Rate Coefficients for OH + NO (+N_2) in the Fall-off Regime and the Impact of Water-Vapor

*Wenyu Sun,[1,#] Jos Lelieveld [1] and John N. Crowley [1] **

[1]Max-Planck-Institute for Chemistry, Division of Atmospheric Chemistry, Hahn-Meitner-Weg 1, 55128 Mainz, Germany

*Email: john.crowley@mpic.de

Present Addresses:

[#]W.S.: Lawrence Livermore National Laboratory, CA, USA (Email: sun39@llnl.gov)

Description of fitting constraints (*Methods 1-5*) and associated Figures (S1-S3)

Different approaches (*Methods 1-5*) were applied when fitting the data to Eqn. (2) and the resulting parameters are provided in **Table S1**, an extension to **Table 2** of the main manuscript.

Method 1.

To reduce the number of variables in the fitting, and also because a relatively small temperature range is covered by the current measurements, we assumed that $k_{1,\infty}$ is independent of the temperature (m = 0). This assumption is reasonable as m is expected to be small and is not accurately defined by the high-pressure data. Indeed, small positive values of 0.3 and 0.1 are used in the IUPAC and NASA evaluations, respectively. When all other parameters ($k_{1,0}^{N2}$, $k_{1,\infty}$, n and F_C) are allowed to vary, the least-squares optimization with Eqn. (3) and Eqn. (4) yields $k_{1,0}^{N2}$ = 7.15×10^{-31} cm^{6}molecule^{-2}s^{-1}, n = 2.35, $k_{1,\infty}$ = 2.02×10^{-11} cm^{3}molecule^{-1}s^{-1}and F_C = 0.69 (*Method 1* in **Table S1**). These parameters, with an R^2 coefficient of 0.9966, can accurately capture all our measurements, as presented in **Fig. S1**.

Method 2.

If we allow m to float during fitting (*Method 2*), *i.e.* assume that $k_{1,\infty}$ is temperature-dependent, the resulting values of $k_{1,0}^{N2}$ (7.14×10^{-31} cm^{6}molecule^{-2}s^{-1}) and F_C (0.67) are very similar to those obtained with *Method 1*, while n (2.88) and $k_{1,\infty}$ (2.20×10^{-11} cm^{3}molecule^{-1}s^{-1}) are larger than the values of *Method 1* by around 10%. The sign of m is however negative and with a relatively large absolute value of 0.92. While the extra fit-variable results in a higher quality of fit (with an R^2 coefficient of 0.9970) the value and sign of m results in an unphysical 40% increase in $k_{1,\infty}$ when the temperature is increased from 273 K to 333 K.

Method 3 and 4

To examine the effects of using the theoretical value of F_C and experimental $k_{1,\infty}$, we fit the data with F_C fixed at 0.81 (*Method 3*) and by fixing $k_{1,\infty}$ at 3.3×10^{-11} cm^{3}molecule^{-1}s^{-1} with m at 0.3 (*Method 4*). The fits obtained in both *Method 3* and *Method 4* are of reasonable quality (See **Table S2)** with R^2 coefficients over 0.996. *Method 3* gives a much smaller value of $k_{1,\infty}$ (1.57×10^{-12} cm^3 molecule^{-1} s^{-1}) with a negative m (-0.53), while *Method 4* (**Fig. 6** in the main text) leads to a significantly lower F_C (0.53), in comparison to the values preferred by IUPAC or obtained through *Methods 1* and *2* in this work.

Method 5.

In *Method 5*, in which both F_C and the high-pressure limiting rate coefficient are set to values reported by IUPAC, the experimental values of k_1 are poorly reproduced, especially in the pressure range below 10^{19} molecule cm^{-3} (as presented in **Fig. S2)**. The disagreement between the measurements and the parameterization obtained through *Method 5* suggests that the high-pressure limiting rate coefficients and the broadening factor recommended by IUPAC, or at least one of the parameters, might not be well defined.

Table S1. Parameterization of k_1 in N_2

	$k_{1,0}^{N2}$ [a]	n	$k_{1,\infty}$ [b]	m	F_C	RSD[c]	Temperature
Method 1	7.15	2.35	2.02	**0.00**	0.69	1.13	273−333 K
Method 2	7.14	2.88	2.20	-0.92	0.67	1.07	273−333 K
Method 3	6.72	2.66	1.57	-0.53	**0.81**	1.15	273−333 K
Method 4	7.24	2.17	**3.30**	**0.30**	0.53	1.21	273−333 K
Method 5	4.77	1.57	**3.30**	**0.30**	**0.81**	3.37	273−333 K
IUPAC	7.4	2.4	3.3	0.3	0.81	--	200−400 K
NASA[d]	7.1	2.6	3.6	0.1	0.6	--	--

Notes: [a] Units of 10^{-31} cm^6 $molecule^{-2}$ s^{-1}, [b] Units of 10^{-12} cm^3 $molecule^{-1}$ s^{-1}, [c] Residual Standard Deviation is defined as $(\Sigma(k_1-k_{1p})^2 / (N-2))^{0.5}$ where k_1 and k_{1p} are the measured and the fitted rate coefficients and N is the total number of data points, the unit is 10^{-13} cm^3 $molecule^{-1}$ s^{-1}. [d] The simplified form of the Troe expression for termolecular reactions used by NASA can be found in the Supplementary Information below. Numbers in bold-type were fixed during fitting.

In summary, the parameterizations by *Method 1* and *Method 4* result in reasonable values of the parameters varied and are preferred in this work over the other methods. A comparison *Method 1* and *Method 4* and the current measurements are presented in **Fig. S3**, and the low pressure range (below 100 Torr) is "zoomed in" in **Fig. S4**. The choice between them depends on the specific objectives. *Method 1*, with a lower residual standard deviation (see **Table S2**), can better reproduce our current measurements (in the fall-off regime) than *Method 4*, but the extrapolated high-pressure limiting rate coefficients bear large uncertainties. For the purpose of modeling the Earth's atmosphere and constraining the fit to the data of the H_2O-N_2 experiments, the accurate characterization of k_1 at low and intermediate pressures is of primary importance and the correct definition of $k_{1,\infty}$ is less essential. However, the differences in fit-quality are small and as *Method 4* incorporates a high-pressure limiting rate coefficient with an experimental basis[1-2] we use the results obtained by *Method 4* when e.g. comparing our data with previous evaluations.

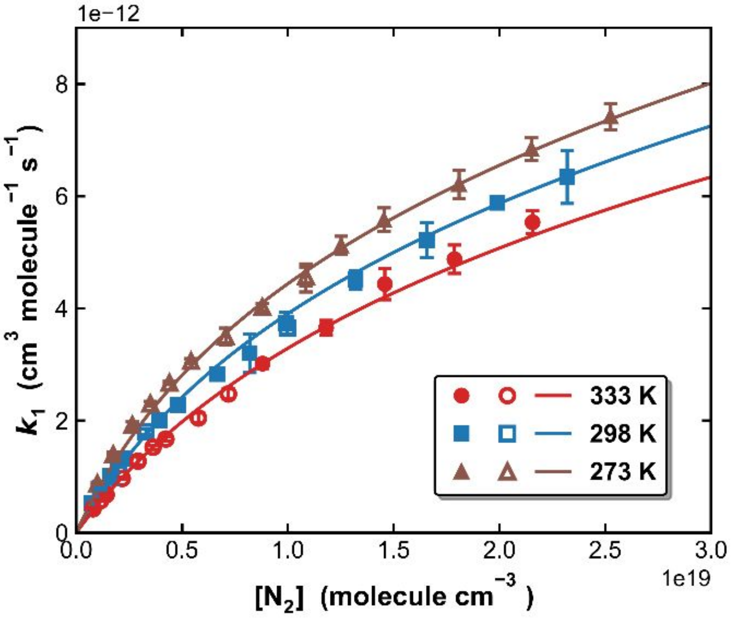

Fig. S1. Measured k_1 (symbols) as a function of [N_2] at 273, 298 and 333 K in this work. The solid lines are the fits of experimental data using *Method 1*.

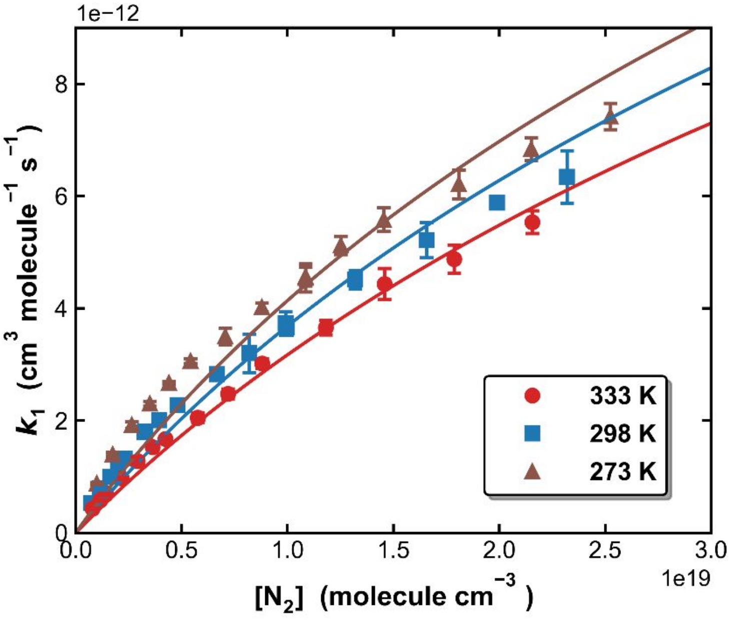

Figure S2. Measured k_1 (symbols) as a function of [N_2] at 273, 298 and 333 K in this work. The solid lines are the fits of experimental data to Eqs. 3 and 4 by fixing $k_{1,\infty}$ at 3.3 ×10^{-11} cm^3 molecule^{-1} s^{-1}, *m* at 0.3 and F_C at 0.81, (*Method 5*) as preferred in the IUPAC parameterization. The fitted $k_{1,0}^{N2}$ and *n* are 4.77×10^{-31} cm^6 molecule^{-2} s^{-1} and 1.57 respectively.

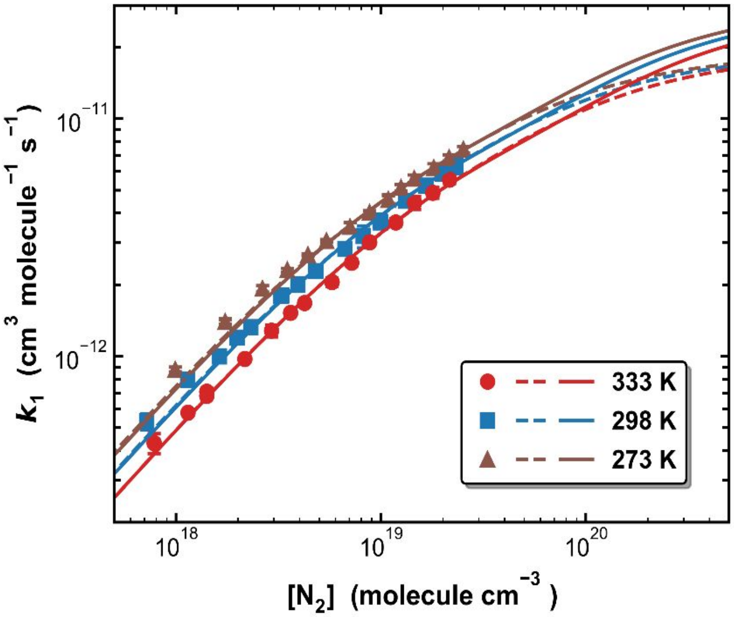

Figure S3. Measured k_1 (symbols) as a function of [N_2] in comparison with parameterizations obtained using *Method 1* (dashed lines) and *Method 4* (solid lines).

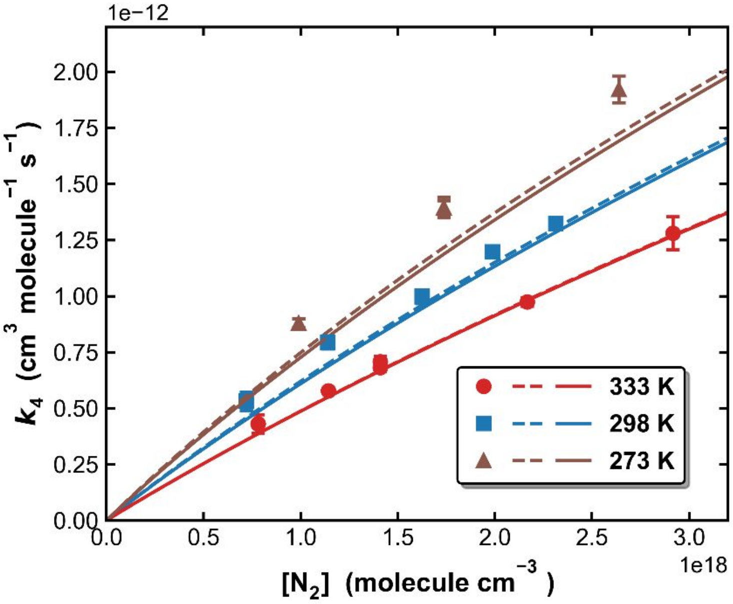

Figure S4. Measured k_1 (symbols) as a function of [N_2] in comparison with parameterizations obtained using *Method 1* (dashed lines) and *Method 4* (solid lines) below 100 Torr.

Comparison of k_1 with evaluations (Figures S5 and S6).

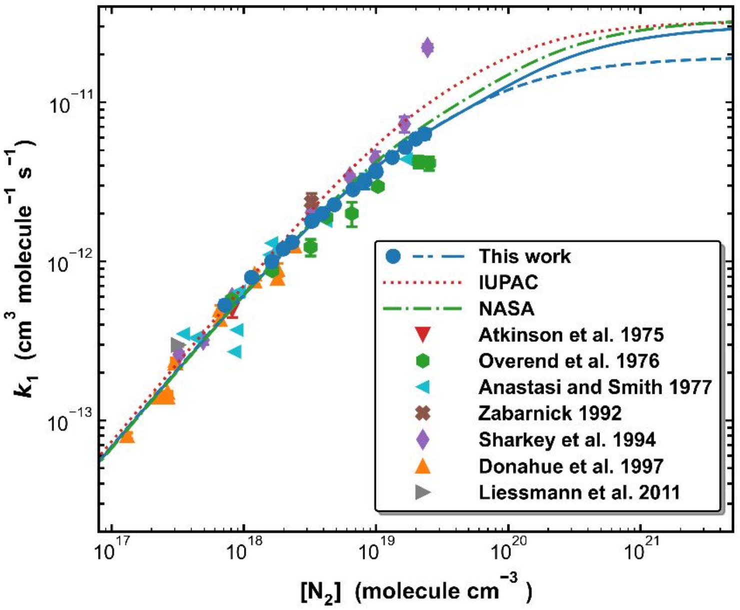

Figure S5. A comparison of measured and parameterized k_1 in N_2 bath gas at around 298 K. The lines are values of k_1 derived from the parameterizations presented in this work using *Method 1* (dashed line) and *Method 4* (solid line) and those by the IUPAC[3] and NASA[4] data-evaluation panels.

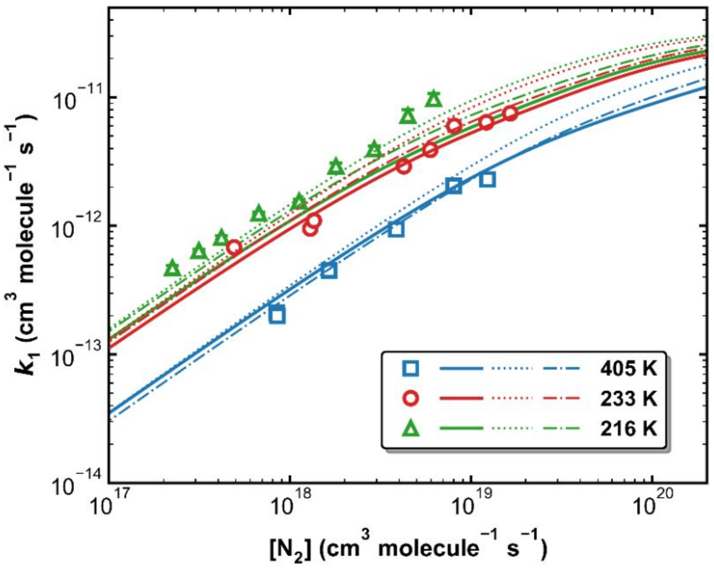

Figure S6. A further comparison for the current parameterization of k_1 (*Method 4*, solid lines) with literature measurements at temperatures of 405, 233 and 216 K. Experimental data at 405 and 233 K are from Anastasi and

Smith,[5] the dataset at 216 K was reported by Sharkey et al.[6] Dotted and dash-dotted lines are values of k_1 calculated using the IUPAC[3] and NASA[4] recommendations, respectively.

NASA parameterization method for termolecular reactions

The NASA evaluation panel[4] uses a simplified form of the Troe expression for termolecular reactions, with

$$k_{NASA}\,(P,T) = \frac{k_0\left(\frac{T}{298\,K}\right)^{-n}[M]}{1+\frac{k_0\left(\frac{T}{298\,K}\right)^{-n}[M]}{k_\infty\left(\frac{T}{298\,\mathrm{K}}\right)^{-m}}}0.6^{\left\{1+\left[log\left(\frac{k_0\left(\frac{T}{298\,\mathrm{K}}\right)^{-n}[M]}{k_\infty\left(\frac{T}{298\,\mathrm{K}}\right)^{-m}}\right)\right]^2\right\}^{-1}} \qquad \text{(S1)}$$

where [M] = molecular density (in molecule cm^{-3}), T is in kelvin. This expression should be used when inputting the "NASA" parameters from **Table 2**.

Parametrization of k_1 in N_2-H_2O bath-gas using different values of F_C for N_2 and H_2O

According to the approach proposed by Burke and Song,[7] the broadening factor for a gas-mixture can also expressed by the weighed sum of the broadening factors in the two individual bath gases, in this case N_2 and H_2O:

$$\log F^{\mathrm{N_2-H_2O}} = X_{\mathrm{N_2}}\log F^{\mathrm{N_2}} + X_{\mathrm{H_2O}}\log F^{\mathrm{H_2O}} \qquad \text{(S2)}$$

where

$$\log F^{\mathrm{N_2}} = \frac{\log F_{\mathrm{C}}^{\mathrm{N2}}}{1+\left[\log\left(\frac{(x_{\mathrm{N2}}k_{1,0}^{\mathrm{N2}}\left(\frac{T}{300\,K}\right)^{-n}+x_{\mathrm{H_2O}}k_{1,0}^{\mathrm{H2O}}\left(\frac{T}{300\,K}\right)^{-o})[M]}{k_{4,\infty}\left(\frac{T}{300\,K}\right)^{-m}}\right)/(0.75-1.27\log F_{\mathrm{C}}^{\mathrm{N2}})\right]^2} \qquad \text{(S3)}$$

$$\log F^{\mathrm{H_2O}} = \frac{\log F_{\mathrm{C}}^{\mathrm{H2O}}}{1+\left[log\left(\frac{(x_{\mathrm{N_2}}k_{1,0}^{\mathrm{N2}}\left(\frac{T}{300\,K}\right)^{-n}+x_{\mathrm{H_2O}}k_{1,0}^{\mathrm{H2O}}\left(\frac{T}{300\,K}\right)^{-o})[M]}{k_{1,\infty}\left(\frac{T}{300\,K}\right)^{-m}}\right)/(0.75-1.27\log F_{\mathrm{C}}^{\mathrm{H2O}})\right]^2} \qquad \text{(S4)}$$

, and the weights for the N_2 and the H_2O terms are characterized by:

$$X_{\mathrm{N_2}} = \frac{x_{\mathrm{N_2}}k_{1,0}^{\mathrm{N2}}\left(\frac{T}{300\,K}\right)^{-n}[M]}{(x_{\mathrm{N_2}}k_{1,0}^{\mathrm{N2}}\left(\frac{T}{300\,K}\right)^{-n}+x_{\mathrm{H_2O}}k_{1,0}^{\mathrm{H2O}}\left(\frac{T}{300\,K}\right)^{-o})[M]} \qquad \text{(S5)}$$

$$X_{\mathrm{H_2O}} = \frac{x_{\mathrm{H_2O}}k_{1,0}^{\mathrm{H2O}}\left(\frac{T}{300\,K}\right)^{-o}[M]}{(x_{\mathrm{N_2}}k_{1,0}^{\mathrm{N2}}\left(\frac{T}{300\,K}\right)^{-n}+x_{\mathrm{H_2O}}k_{1,0}^{\mathrm{H2O}}\left(\frac{T}{300\,K}\right)^{-o})[M]} \qquad \text{(S6)}$$

where F_C^{N2} and F_C^{H2O} are the broadening factors at the center of the fall-off curves of N_2 and H_2O, respectively.

References

1. Forster, R.; Frost, M.; Fulle, D.; Hamann, H. F.; Hippler, H.; Schlepegrell, A.; Troe, J. High pressure range of the addition of HO to HO, NO, NO_2, and CO. I. Saturated laser induced fluorescence measurements at 298 K. *J. Chem. Phys.* **1995,** *103*, 2949-2958.
2. Fulle, D.; Hamann, H. F.; Hippler, H.; Troe, J. Temperature and pressure dependence of the addition reactions of HO to NO and to NO_2. IV. Saturated laser-induced fluorescence measurements up to 1400 bar. *J. Chem. Phys.* **1998,** *108*, 5391-5397.
3. IUPAC Task Group on Atmospheric Chemical Kinetic Data Evaluation, (Ammann, M., Cox, R.A., Crowley, J.N., Herrmann, H., Jenkin, M.E., McNeill, V.F., Mellouki, A., Rossi, M. J., Troe, J. and Wallington, T. J.). Last access Sept. 2021. http://iupac.pole-ether.fr/index.html.
4. Burkholder, J.; Sander, S.; Abbatt, J.; Barker, J.; Cappa, C.; Crounse, J.; Dibble, T.; Huie, R.; Kolb, C.; Kurylo, M. *Chemical kinetics and photochemical data for use in atmospheric studies; evaluation number 19*; Jet Propulsion Laboratory, National Aeronautics and Space Administration: 2020.
5. Anastasi, C.; Smith, I. W. M. Rate measurements of reactions of OH by resonance absorption. Part 6.—Rate constants for OH + NO (+M)→ HNO_2 (+M) over a wide range of temperature and pressure. *J. Chem. Soc. Faraday Trans II* **1978,** *74*, 1056-1064.
6. Sharkey, P.; Sims, I. R.; Smith, I. W. M.; Bocherel, P.; Rowe, B. R. Pressure and temperature dependence of the rate constants for the association reaction of OH radicals with NO between 301 and 23 K. *J. Chem. Soc., Faraday Trans.* **1994,** *90*, 3609-3616.
7. Burke, M. P.; Song, R. Evaluating mixture rules for multi-component pressure dependence: H+O_2 (+M)=HO_2 (+M). *Proc. Comb. Inst.* **2017,** *36*, 245-253.
